# Effects of Light Quality and Intensity on Diurnal Patterns and Rates of Photo-Assimilate Translocation and Transpiration in Tomato Leaves

**DOI:** 10.3389/fpls.2018.00756

**Published:** 2018-06-04

**Authors:** Jason Lanoue, Evangelos D. Leonardos, Bernard Grodzinski

**Affiliations:** ^1^Department of Plant Agriculture, University of Guelph, Guelph, ON, Canada; ^2^Harrow Research and Development Centre, Agriculture and Agri-Food Canada, Harrow, ON, Canada

**Keywords:** carbon export, light-emitting diode (LED), light quality, water-use-efficiency (WUE), photosynthesis, tomato, translocation, transpiration

## Abstract

Translocation of assimilates is a fundamental process involving carbon and water balance affecting source/sink relationships. Diurnal patterns of CO_2_ exchange, translocation (carbon export), and transpiration of an intact tomato source leaf were determined during ^14^CO_2_ steady-state labeling under different wavelengths at three pre-set photosynthetic rates. Daily patterns showed that photosynthesis and export were supported by all wavelengths of light tested including orange and green. Export in the light, under all wavelengths was always higher than that at night. Export in the light varied from 65–83% of the total daily carbon fixed, depending on light intensity. Photosynthesis and export were highly correlated under all wavelengths (*r* = 0.90–0.96). Export as a percentage of photosynthesis (relative export) decreased as photosynthesis increased by increasing light intensity under all wavelengths. These data indicate an upper limit for export under all spectral conditions. Interestingly, only at the medium photosynthetic rate, relative export under the blue and the orange light-emitting diodes (LEDs) were higher than under white and red-white LEDs. Stomatal conductance, transpiration rates, and water-use-efficiency showed similar daily patterns under all wavelengths. Illuminating tomato leaves with different spectral quality resulted in similar carbon export rates, but stomatal conductance and transpiration rates varied due to wavelength specific control of stomatal function. Thus, we caution that the link between transpiration and C-export may be more complex than previously thought. In summary, these data indicate that orange and green LEDs, not simply the traditionally used red and blue LEDs, should be considered and tested when designing lighting systems for optimizing source leaf strength during plant production in controlled environment systems. In addition, knowledge related to the interplay between water and C-movement within a plant and how they are affected by environmental stimuli, is needed to develop a better understanding of source/sink relationships.

## Introduction

Different light quality, as well as intensity, provided by wavelength specific light-emitting diodes (LEDs) have been shown to affect both leaf CO_2_ fixation and transpiration in tomato (Lanoue et al., 2017). In addition to photosynthesis, export is also a key process of a source leaf defining its strength. Export involves both the movement of carbon and water through the translocation conduit. For growth of a plant to actually occur, the photo-assimilates synthesized in leaves must be exported to growing sink tissues. Up to 80% of fixed carbon is exported via phloem translocation in the day and night periods (Grange, 1985; Geiger and Servaites, 1994; Lemoine et al., 2013). Importantly in studies using attached leaves, it is clear that most export occurs in the day-time rather than during subsequent night periods (Kalt-Torres et al., 1987; Leonardos and Grodzinski, 2000; Leonardos et al., 2003). Translocation at night can involve the breakdown of starch as well as mobilization of sugars (Geiger and Servaites, 1994; Lemoine et al., 2013). An important consideration regarding source/sink flow of assimilates is that the key processes which regulate translocation are not merely the classical enzymatic pathways of sucrose and starch metabolism (Geiger and Servaites, 1994; Lemoine et al., 2013), but also steps involving many transporters and temporary storage sites (Farrar and Farrar, 1986; Lemoine et al., 2013; Osorio et al., 2014).

Notably, at ambient CO_2_ conditions and high light intensity, a high correlation is observed between photosynthesis and export in many C_3_ and C_4_ species (Grodzinski et al., 1998; Leonardos and Grodzinski, 2000). In tomato, a sucrose exporter, sucrose concentration and C-export rates are highly correlated across a wide range of photosynthetic rates (Ho, 1976). An increase of C-fixation of 1 mg C dm^-2^ h^-1^ results in an increase in export rate of 0.59 mg C dm^-2^ h^-1^ quantified using differential leaf weight analysis (Ho, 1976). Although much is known about sugar synthesis during photosynthesis, little is known about how light quality alters translocation patterns of attached source leaves.

An early study of translocation using detached sugarcane leaves at low light levels indicated that green light caused a decrease in translocation compared to red and blue (Hartt, 1966). Furthermore, in a similarly designed ^14^CO_2_ pulse-chase experiment performed with intact parsnip leaflets, no differences in translocation rates under different light spectra were observed (Hoddinott and Gorham, 1975). In order to deploy new LED technology for controlled environments the role of spectral quality on gas exchanges (i.e., of both CO_2_ and H_2_O) and export functions of the source leaf are required.

The inter-play between long-distance assimilate movement via the phloem (Münch, 1930) and the movement of H_2_O within the xylem via transpiration (Dixon and Joy, 1894) is still poorly understood (Windt et al., 2006; Nikinmaa et al., 2013). Although studies with woody species using MRI or theoretical modeling have suggested that transpiration rate affects export (Windt et al., 2006; Nikinmaa et al., 2013), there has been very little experimental data linking H_2_O and CO_2_ exchanges with the mobility of H_2_O and assimilates via translocation using intact herbaceous plants (Johnson et al., 1992). Furthermore, our understanding of carbon fixation and translocation suffers from the fact that very little data exists where researchers have used intact, attached leaves so that tissue turgor and metabolism have both not been jeopardized.

Regulation of plant growth and development is fine-tuned by photoreceptors (i.e. cryptochrome, phytochrome, etc.) responding to light of different spectral quality throughout the day (Chen et al., 2004). There is a long-distance interplay between source and sink interactions that occurs throughout the day. It is well known that growth patterns of sink organs change in response to environmental stimuli including light intensity and quality (Heuvelink, 1989; Bertram and Karlsen, 1994; Lin, 2000; Nozue and Maloof, 2006; Liu et al., 2012). For example, long term acclimation to blue light causes tomato plants to be shorter (Lin, 2000; Liu et al., 2012). Thus, the problem with studying export from source leaves of plants which have been acclimated to wavelength specific lighting, is that one is comparing source/sink relationships in plants with different morphology and anatomy. Therefore, information about the effect of spectral quality on export is required before moving to plants acclimated to different abiotic stresses such as those occurring in greenhouses during the implementation of inter-canopy lighting (Hao et al., 2012; Gomez and Mitchell, 2014).

With such a large gap existing in our understanding of source-to-sink metabolism with regards to light quality, we set out to challenge intact, attached source leaves with wavelength specific lighting and measure diurnal patterns of gas exchanges and C-export. We hypothesize, that, due to the complexity of the C-export pathway within the source leaf, the effects of wavelength specific lighting can be manifested independently of the effects of light quality on the primary photosynthetic reactions in the chloroplast. We developed a novel methodology to study diurnal patterns of photosynthesis and export, using non-acclimated source leaves under maximized sink demand. We used a steady-state ^14^CO_2_ labeling technique and exposed mature tomato source leaves to LEDs with differing spectral quality. To better compare the diurnal C-export patterns, we purposely established three different photosynthetic rates at the beginning of the photoperiod to drive similar initial CO_2_ influx rates among all lights. We also examined if there was any link between intact source leaf transpiration and C-export.

## Materials and Methods

### Plant Material and Growth Conditions

Seeds of *Solanum lycopersicum* cv. ‘Bonny Best’ from William Dam Seeds (Dundas, ON, Canada) were sown into 60 cavity potting trays in Sungro professional growing mix #1 (Soba Beach, AB, Canada) under a clear plastic lid to maintain a high relative humidity (RH; ∼85%) and placed in a growth chamber (GC-20 Bigfoot series, Biochambers, Winnipeg, MB, Canada) at 22/18°C (day/night) with a 16/8 h photoperiod and 200 ± 25 μmol m^-2^ s^-1^ of photosynthetically active radiation (PAR) from compact fluorescence lights (CFLs; Sylvania Pentron 841 HO Ecologic, Wilmington, MA, United States; Supplementary Figure 1). After germination, lids were removed and plants were grown at 65 ± 10% RH, ambient CO_2_ (400 μL L^-1^), and 300 ± 25 μmol m^-2^ s^-1^ PAR at canopy level. Plants were watered with fertilizer (24-8-16; Miracle Gro^TM^, Marysville, OH, United States).

Three days prior to ^14^CO_2_ feeding, the photoperiod was changed from 16 h/8 h to 12 h/12 h. The extended night period was done in order to reduce the sucrose pools within the leaves to ensure isotopic equilibrium would be reached quickly during the ^14^C labeling experiments without harming the photosynthetic capability (Gibon et al., 2004).

### Leaf Gas Exchange and ^14^C-Export

A steady-state ^14^CO_2_ labeling technique was employed (Geiger and Fondy, 1979) using a custom-made leaf gas exchange/^14^C labeling system previously as described in Leonardos et al. (1996, 2003). Plants 30–35 days after germination were illuminated with white CFLs for 30 min at 300 ± 25 μmol m^-2^ s^-1^ PAR at canopy level in order to prime all photosystems (Supplementary Figure 1). Four plants for each experimental run were then transferred to the ^14^C system where the most distal leaflet on the 5th highest leaf was placed in a leaf chamber and sealed. Each of the four leaf chambers included a circulating water jacket for temperature control, a glass window on the top to allow light to illuminate the leaf, and a Geiger-Muller (GM) detector (model 7231, LND Inc., Oceanside, NY, United States) underneath the entire leaf area enclosed in the chamber (16 cm^2^) for radioactivity monitoring.

The leaf was illuminated with one of seven spectra from custom LEDs provided by Lighting Science Group Company (LSGC; Warwick, RI, United States) including white (W), red-blue (RB), red-white (RW), red (R), blue (B), orange (O), and green (G) (Supplementary Figure 1). Photosynthetic rates achieved in the ^14^C system were set to ∼12, 8, or 4 μmol m^-2^ s^-1^, respectively, at the start of the experiment before ^14^CO_2_ was added, by adjusting light levels for each leaf chamber/light treatment using previous data (Lanoue et al., 2017). The rates of ∼12, 8, and 4 μmol m^-2^ s^-1^ represent, by design, photosynthetic conditions that were near saturating (high), medium, and low ranges respectively. Each lighting treatment and photosynthetic rate was randomized daily to ensure there was no chamber bias. The experimental design was specific to our primary objective to compare daily export patterns under the different spectra, but at very similar CO_2_ influx rates.

Only the source ^14^C-fed leaf was illuminated. The remainder of the plant was left in the dark to maximize sink demand and therefore maximize source activity and C-export from the illuminated leaf. During the period of illumination, chambers were set to 22°C, 50–60% RH and 405 ± 10 μL L^-1^ of CO_2_ at an air flow rate of 500 cm^3^ min^-1^ per chamber.

Radiolabelled CO_2_ (^14^CO_2_) was generated in a large gas tight syringe by reacting either NaH^14^CO_3_ with 30% H_2_SO_4_ or Ba ^14^CO_3_ with 30% HCl. ^14^CO_2_ was drawn into a 60 mL syringe and loaded onto a syringe pump (PHD 2000 Infusion, Harvard Apparatus, Cambridge, MA, United States). Once gas exchange was deemed to be steady (approximately 30 min after putting the leaf into a chamber), ^14^CO_2_ was injected into the total air stream (2250 cm^3^ min^-1^) at 4 mL h^-1^.

Leaves were illuminated for 15 h during steady-state ^14^CO_2_ labeling (7:00:00–22:00:00). Net carbon exchange rate (NCER) and transpiration rates were obtained by an infrared gas analyzer (IRGA; Li-COR CO_2_/H_2_O Gas analyzer 7000, Lincoln, NE, United States). Day-time C-export was calculated as the difference between NCER measured by the IRGA and the ^14^C retention measured by the GM detector. The ^14^C retention measured by the GM detector was corrected for the specific activity in the air stream (54.75–136.83 Bq μmol^-1^ C) and the GM detector efficiency (0.0064–0.0278) both of which remained steady throughout the day (i.e., each experimental run), but changed between runs.

After 15 h, leaves were either removed from the system for analysis of the products made during the feed period under the LEDs, or subject to an 8 h chase period in the dark while ^14^CO_2_ was not injected. During this night-time chase period, temperature was lowered to 18°C and the flow rate was reduced to 150 cm^3^ min^-1^ per leaf chamber. The respired air was collected in chamber specific gas traps containing 40mL 20% KOH and the respired ^14^C was determined via liquid scintillation counting. NCER during the dark period (respiration rate) was again obtained by the IRGA as were transpiration rates. Night-time C-export was calculated as the difference between ^14^C-retention measured by the GM detector and the respiration rate determined from the radioactivity in the KOH traps.

In order to determine the fate of all ^14^C assimilated, a carbon budget analysis was performed by integrating the day-time rates of C-fixation and export as well as the night-time export and respiration values. Each of these values were then expressed as a percentage of total fixed carbon to allow for analysis of relative day-time and night-time export.

### C-Partitioning

Immediately after each experimental run, leaves were taken out of the chambers in order to determine ^14^C amounts in various forms (i.e., sucrose, starch). The area of the leaf enclosed by the chamber was imaged to determine leaf area then extracted three times using 80% boiling ethanol for 20–30 min, leaving an ethanol soluble fraction and ethanol insoluble fraction. Ethanol soluble fractions were then dried and suspended in a mixture of water and 99% chloroform (3:2 v/v), agitated, and centrifuged at 11,000 RPM to separate a water soluble fraction (primarily sugars) from chloroform soluble leaf components (chlorophyll, lipids, etc.). Ethanol insoluble fractions (primarily starch) were oven dried at 70°C for 48 h, dry ground and suspended in 80% ethanol. ^14^C content of each fraction was determined using liquid scintillation counting.

### Statistical Analysis

#### Diurnal Patterns of Gas Exchange and Export

For each point/bar on a graph presented, an average ± the standard error are represented for the following number of replicates (*n*). During the day-time of high photosynthetic rate experiments starting at ∼12 μmol m^-2^ s^-1^, the number of replicates for W was *n* = 14, for RB was *n* = 11, for RW was *n* = 10, for R was *n* = 14, for B was *n* = 11, for O was *n* = 8, and for G was *n* = 8. During the subsequent 8 h dark period of the same experiment, the number of replicates for W was *n* = 7, for RB was *n* = 5, for RW was *n* = 5, for R was *n* = 7, for B was *n* = 6, for O was *n* = 2, and for G was *n* = 2.

During the day-time of medium photosynthetic rate experiments starting at ∼8 μmol m^-2^ s^-1^, the number of replicates for W was *n* = 19, for RB was *n* = 15, for RW was *n* = 17, for R was *n* = 21, for B was *n* = 23, for O was *n* = 11, and for G was *n* = 12. During the subsequent 8h dark period of the same experiment, the number of replicates for W was *n* = 8, for RB was *n* = 7, for RW was *n* = 4, for R was *n* = 13, for B was *n* = 13, for O was *n* = 6, and for G was *n* = 5.

During the day-time of low photosynthetic rate experiments starting at ∼4 μmol m^-2^ s^-1^, the number of replicates for W was *n* = 15, for RB was *n* = 11, for RW was *n* = 9, for R was *n* = 17, for B was *n* = 15, for O was *n* = 11, and for G was *n* = 9. For the subsequent 8 h dark period of the same experiment, the number of replicates for W was *n* = 7, for RB was *n* = 4, for RW was *n* = 4, for R was *n* = 5, for B was *n* = 8, for O was *n* = 5, and for G was *n* = 5.

All mean comparisons were done with “Contrast” statements in SAS using a Student’s *t*-test with a *p* < 0.05 indicating a significant difference.

#### Correlation Analysis

For correlation analyses displayed in **Figure 6**, hourly data from all export experimentation was pooled (*n* = 3653). The first 2 h of every experiment were excluded from the correlation analysis due to the fact that isotopic equilibrium was not met during this time period. For correlation analysis involving the last hour’s export rate and end of photoperiod soluble sugar concentrations, *n* = 153. The Pearson’s correlation coefficient was classified using guidelines specified by Mukaka (2012).

All statistics were performed using SAS studio 3.5.

## Results

### Diurnal Patterns and Correlation of NCER, C-Export, and Leaf Soluble Sugar Content

**FIGURE 1:**
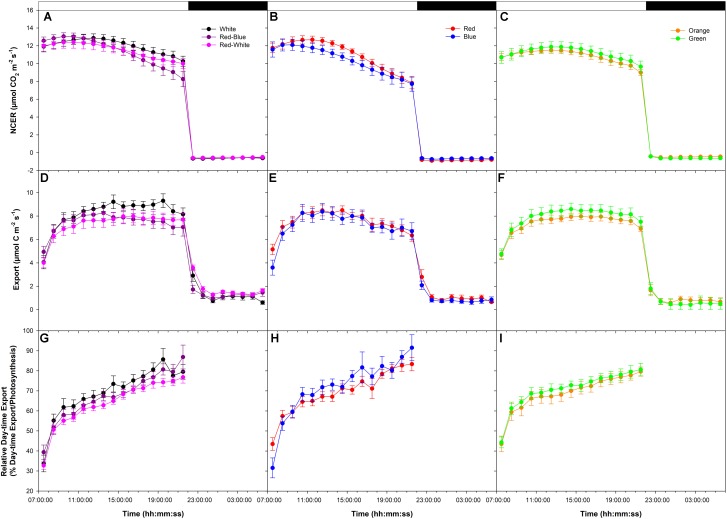
Daily patterns of leaf net carbon exchange rate (NCER) and export at an initial high photosynthetic rate of ∼12 μmol m^-2^ s^-1^ as affected by different spectral qualities. The NCER **(A–C)**, export **(D–F)**, and relative export (export as a percentage of photosynthesis; **G–I**) exposed to mixed light-emitting diodes (LEDs), red-blue (RB), white (W), and red-white (RW) are shown in **A,D,G**. The NCER, export, and relative export from R and B are shown in **B,E,H** while O and G are shown in **C,F,I** respectively.

An initial high photosynthetic rate of ∼12 μmol m^-2^ s^-1^ was established at the start of the photoperiod under all LEDs during the export experiment at high photosynthesis (W, RB, and RW **Figure 1A**; R and B **Figure 1B**; O and G **Figure 1C**). The photosynthetic rate decreased in W, RB, RW, R, and B treatments at about 14:00:00 (**Figures 1A,B**) with the decrease being greater under the monochromatic R and B LEDs (**Figure 1B**). The photosynthetic rate in the O and G LED treatments remained similar throughout the day (**Figure 1C**). Dark respiration rates following the period of illumination were similar (**Figures 1A–C**).

Day-time export rates rose during the beginning of the day under all LEDs (**Figures 1D–F**). During the rest of the illumination period with W, RB, RW, O, and G export remained steady (**Figures 1D,F**), whereas under R and B, export trended downward following the photosynthetic pattern (**Figure 1E**). Night-time export rates were similar following all LED treatments (**Figures 1D–F**).

When day-time export was expressed as a percentage of photosynthesis, similar patterns were observed under all light treatments (**Figures 1G–I**). Relative day-time export increased throughout the day under all LED treatments (**Figures 1G–I**).

**FIGURE 2:**
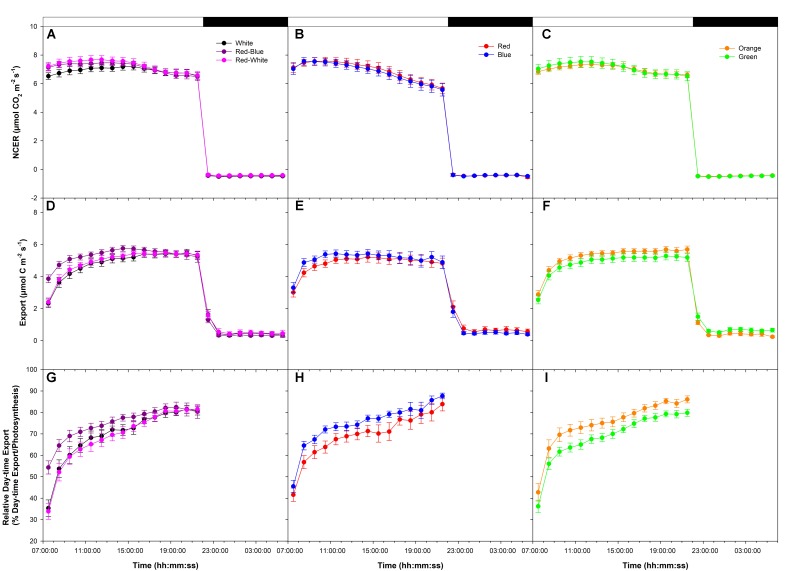
Daily patterns of leaf NCER and export at an initial medium photosynthetic rate of ∼8 μmol m^-2^ s^-1^ as affected by different spectral qualities. The NCER **(A–C)**, export **(D–F)**, and relative export (export as a percentage of photosynthesis; **G–I**) exposed to mixed LEDs system, RB, W, and RW are shown in **A,D,G**. The NCER, export, and relative export from R and B are shown in **B,E,H** while O and G are shown in **C,F,I** respectively.

An initial medium photosynthetic rate of ∼8 μmol m^-2^ s^-1^ was established at the start of the photoperiod under all LEDs in a following export experiment at medium photosynthesis (W, RB, and RW **Figure 2A**; R and B **Figure 2B**; O and G **Figure 2C**). Similar to the high photosynthetic export experiment (**Figure 1**), photosynthetic rates trended downward in all treatments around 16:00:00, more so in the R and B treatments (**Figures 2A–C**). Dark respiration rates were similar following all LED treatments (**Figures 2A–C**).

Export rates from leaves illuminated with W, RB, and RW increased to a maximum between 16:00:00–18:00:00 and remained steady thereafter until the end of the photoperiod (22:00:00; **Figure 2D**). Leaves illuminated with B light reached a maximum export rate at ∼10:00:00 then declined slightly thereafter (**Figure 2E**). Leaves illuminated with R light reached a maximum export rate around 12:00:00 then remained steady until the end of the photoperiod (**Figure 2E**). Export under O and G increased until 14:00:00 and was sustained at a high rate throughout the remainder of the photoperiod (**Figure 2F**).

Under all lights, relative day-time export was seen to increase throughout the photoperiod (**Figures 2G–I**). Interestingly, from 9:00:00 to 12:00:00, leaves illuminated with RB, B, and O LEDs showed a higher relative export rate than did leaves illuminated with W, RW, R, and G LEDs (**Figures 2G–I**). Illumination with O and G light produced a similar, high relative export throughout the photoperiod (**Figure 2I**).

**FIGURE 3:**
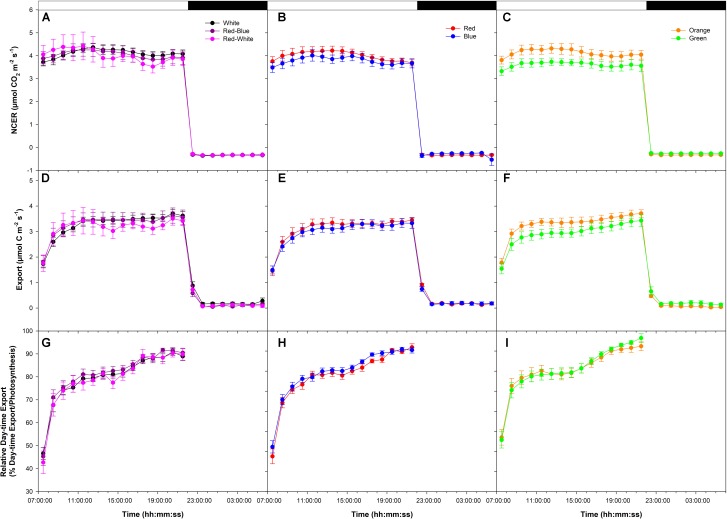
Daily patterns of leaf NCER and export at an initial low photosynthetic rate of ∼4 μmol m^-2^ s^-1^ as affected by different spectral qualities. The NCER **(A–C)**, export **(D–F)**, and relative export (export as a percentage of photosynthesis; **G–I**) exposed to mixed LEDs system, RB, W, and RW are shown in **A,D,G**. The NCER, export, and relative export from R and B are shown in **B,E,H** while O and G are shown in **C,F,I** respectively.

An initial low photosynthetic rate of ∼4 μmol m^-2^ s^-1^ was established at the start of the photoperiod under all LEDs in a final export experiment at low photosynthesis (W, RB, and RW **Figure 3A**; R and B **Figure 3B**; O and G **Figure 3C**). NCER remained stable throughout the day/night period under LEDs (**Figures 3A–C**). Similar to NCER, both day/night export rates were similar under all LEDs (**Figures 3D–F**). It is noteworthy for all photosynthetic rate experiments that day-time export rates were significantly higher than night-time export rates (**Figures 1D–F**, **2D–F**, **3D–F**).

Relative export under all LEDs increased during the morning hours and became steady until 16:00:00 (**Figures 3G–I**). At 16:00:00, under all LEDs, a noticeable increase in relative export was observed which persisted until the end of the photoperiod (**Figures 3G–I**).

**FIGURE 4:**
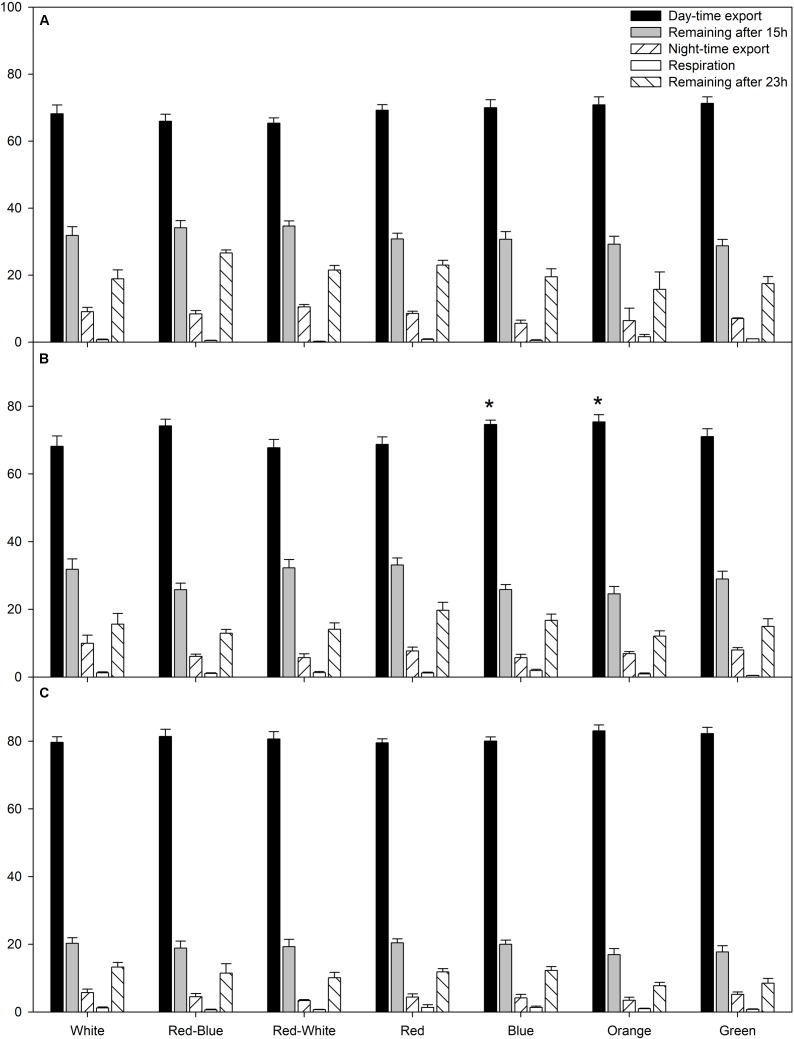
A summary of carbon allocation at high **(A)**, medium **(B)**, and low **(C)** photosynthetic rates to day-time export, storage in the leaf at the end of the photoperiod, night-time export, night-time respiration, and storage in the leaf after 23 h pulse and chase experiment. Carbon allocation is expressed as a percentage of total carbon fixed during the photoperiod. A statistical difference (*p* < 0.05) in the percentage of day-time export between an LED treatment and the W LED control is indicated with an asterisk (^∗^).

The average relative amount of newly fixed carbon from all photosynthetic rate experiments (**Figures 1–3**) which had been either exported, respired, or remained in the leaf after 15 or 23 h are displayed in **Figure 4**. During the experiments at both high and low photosynthetic rates, export during the day and night, as well as the percentage of carbon remaining in the leaf after a 15 h illumination period and subsequent 8 h dark period was similar under all LED treatments (**Figures 4A,C**).

Interestingly, only at the medium photosynthetic rate, leaves illuminated with B and O LEDs produced a higher percentage of day-time export than leaves exposed to the control W LED (**Figure 4B**). Furthermore, leaves illuminated with B and O LEDs also produced a higher percentage of day-time export than leaves exposed to RW LEDs (**Figure 4B**).

**FIGURE 5:**
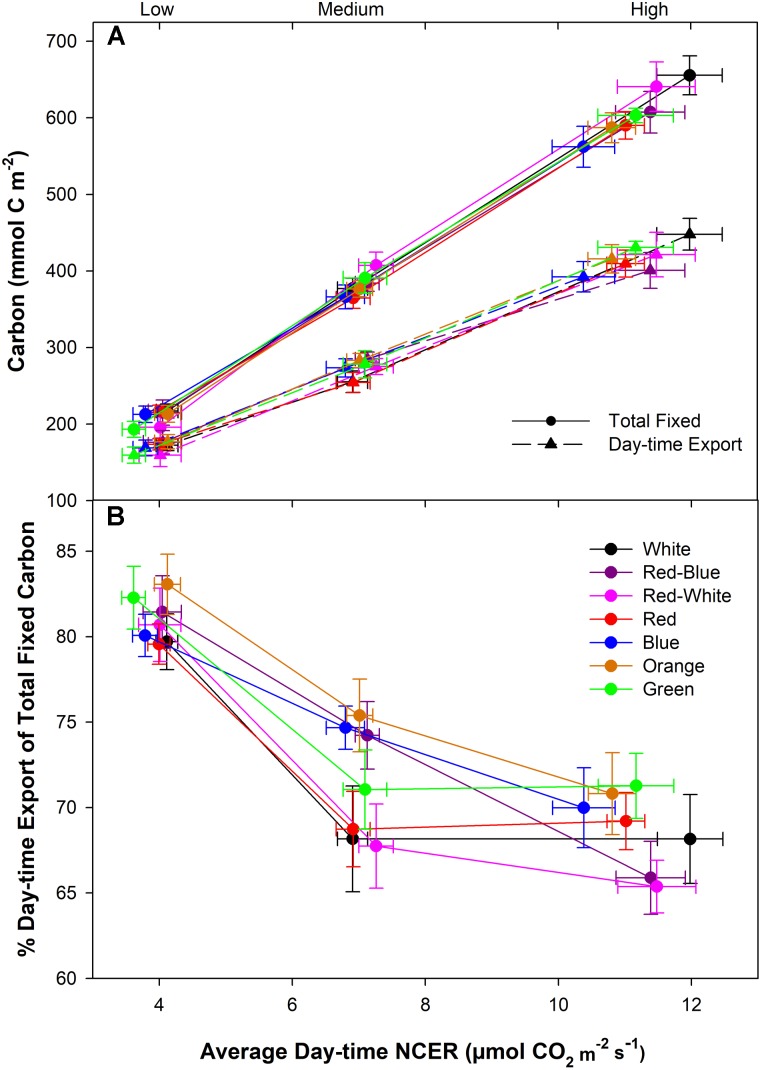
Total carbon fixed and exported during the photoperiod under low, medium, and high photosynthetic rates from leaves that were illuminated by various LED systems **(A)**. **B** illustrates the relationship between export and photosynthesis during the day-time under different LED treatments. Relative export during the day-time was expressed as the percentage of the total fixed carbon.

Under all spectral conditions tested, including the O and G, as the photosynthetic rate was raised by increasing light intensity, the amount of day-time C-export also increased (**Figure 5A**). However, the slope of day-time export did not show the same extent of increase as did the amount of total fixed carbon under all wavelengths (**Figure 5A**). The relationship between total fixed carbon and day-time export is evident in **Figure 5B**, indicating a higher percentage of relative day-time export during the low photosynthetic experiments compared to both medium and high photosynthetic rates. The differences in day-time export under the different spectra at the middle photosynthetic rate noted in **Figure 4B** above are highlighted in **Figure 5B** that shows an interesting separation due to light quality.

**FIGURE 6:**
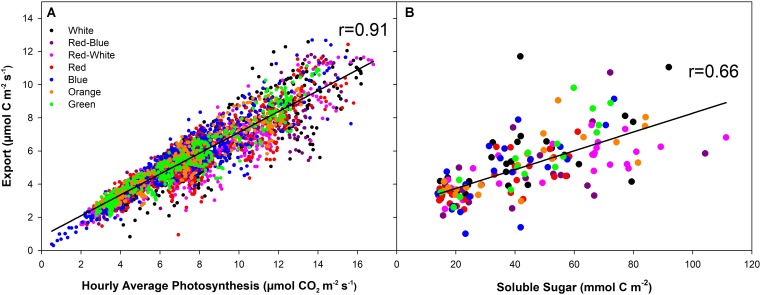
Correlation analysis between hourly averages of carbon fixation and export under illumination with LEDs of different spectral qualities and intensities **(A)**. **B** displays the correlation between the average export during the last hour of illumination and the soluble sugar in the leaf at the end of the illumination period. The solid black line (–) indicates the linear regression line within each dataset.

At all light intensities, a very strong correlation between photosynthesis and export was observed (*r* = 0.91; **Figure 6A**) and independent of the spectral quality the leaf was illuminated with (**Table 1**). A strong correlation (*r* = 0.66) was also observed between the last hour day-time export and end of photoperiod soluble sugar content in the leaf (**Figure 6B**). A moderate to strong correlation between average day-time export and end of photoperiod soluble sugars was observed under each of the lights tested (*r* = 0.52–0.87; **Table 1**). Notably, when leaves were illuminated with a G LED, the strongest correlation between export and soluble sugar content was obtained (*r* = 0.87; **Table 1**).

**Table 1:** Summary of wavelength specific correlation coefficients (*r*) for photosynthesis vs. export (**Figure 6A**), and export vs. soluble sugars (**Figure 6B**).

LED treatment	White	Red-blue	Red-white	Red	Blue	Orange	Green
Photosynthesis vs. Export	0.91	0.92	0.90	0.91	0.93	0.94	0.96
Soluble sugar vs. export	0.61	0.52	0.75	0.74	0.72	0.77	0.87

Our results show the diurnal patterns of C-fixation and export at three pre-established photosynthetic rates and different LED treatments. Of note, within each experimentation, only the intact source leaf was illuminated by an LED while the rest of the plant was kept in darkness to maximize sink demand. Also, plants used were all of similar size and sink demand. Thus, results presented are likely the result of the source leaf environment, specifically light quality and intensity.

### Diurnal Patterns of Leaf Stomatal Conductance, Transpiration Rate, and Water-Use-Efficiency

**FIGURE 7:**
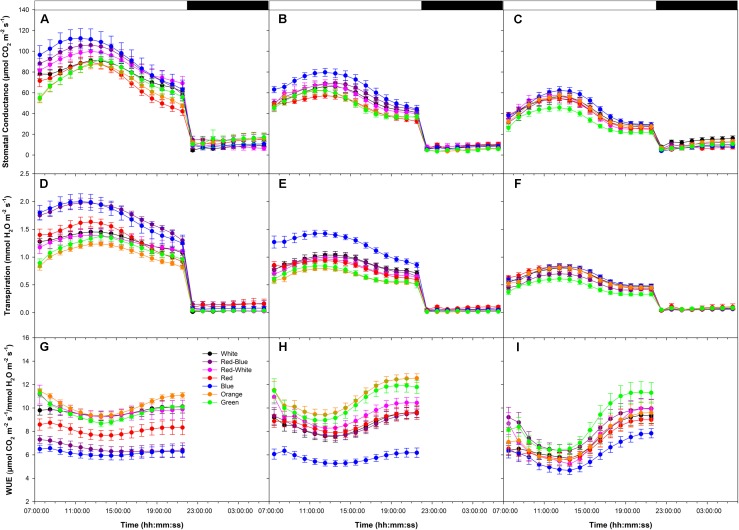
Diurnal patterns of stomatal conductance **(A–C)**, transpiration rate **(D–F)**, and WUE **(G–I)** of leaves during the light period under various wavelength specific LEDs. **A,D,G** show these values from export experiments done at an initial high photosynthetic level of ∼12 μmol m^-2^ s^-1^. Panels **B,E,H** show these values from export experiments done at an initial medium photosynthetic level of ∼8 μmol m^-2^ s^-1^. **C,F,I** show these values from export experiments done at an initial low photosynthetic level of ∼4 μmol m^-2^ s^-1^.

During all photosynthetic rate experiments, day-time stomatal conductance and transpiration rates were higher than night-time rates (**Figures 7A–F**). Stomatal conductance and transpiration rates increased until mid-day and decline thereafter (**Figures 7A–F**). During all photosynthetic rate experiments, leaves illuminated with B LEDs produced the highest stomatal conductance and transpiration rates (**Figures 7A–F**). Day-time patterns of water-use-efficiency (WUE) reached a minimum in all experiments during the middle of the photoperiod then increased thereafter (**Figures 7G–I**). Of note, during all photosynthetic rate experiments, leaves exposed to B light produced the lowest WUE, while leaves exposed to O and G produced among the highest (**Figures 7G–I**).

## Discussion

### The Effect of Wavelength Specific LEDs on Photosynthesis and Export

Under all LED treatments at all photosynthetic levels, photosynthesis and export was sustained throughout the photoperiod (**Figures 1–3**). Consistent with previous studies, a high correlation between photosynthesis and export (*r* = 0.91) as well as export and leaf soluble sugar content (*r* = 0.66) were determined (Grodzinski et al., 1998; Leonardos and Grodzinski, 2000). Day-time export was always greater than night-time export under all conditions, consistent with previous studies using natural sunlight (Kalt-Torres et al., 1987) or artificial, multi-spectrum metal halide lights (**Figures 1–3**; Leonardos et al., 2003).

Importantly, leaves exposed to G produced similar photosynthetic and export rates when compared to other LED treatments. These results add to a growing consensus among literature indicating the ability of plants to function properly under G light (**Figures 1–3**; Sun et al., 1998; Terashima et al., 2009; Wang and Folta, 2013). Furthermore, results displaying the diurnal patterns of photosynthesis and C-export of leaves exposed to O light introduce novel information pertaining to the function of the primary CO_2_ gas exchange and carbon metabolism processes (**Figures 1–3**). The emphasis has traditionally been on R and B LEDs because of their central role in the activation of the chlorophyll reaction centers (Hogewoning et al., 2010). To our knowledge, the commercial application of O and G LEDs for illumination in controlled environment systems has received little attention but, clearly warrants further examination.

Relative export increased throughout the photoperiod under all conditions (**Figures 1–3**). Tomatoes are known to store and re-mobilize sucrose in vacuoles throughout the day (Osorio et al., 2014). Similarly, in barley, vacuolar sucrose decreases in the afternoon in support of C-export (Farrar and Farrar, 1986). Starch synthesis and degradation occurs simultaneously during the light period (Stitt and Heldt, 1981; Zeeman et al., 2004; Osorio et al., 2014). Thus, the increase in relative export during the afternoon hours observed, specifically at the low photosynthetic rates (**Figures 3G–I**), is consistent with the remobilization of temporarily stored assimilates.

During experimentation at the medium photosynthetic rate, day-time export from B and O illuminated leaves was higher than from leaves illuminated with W or RW (**Figure 4B**). These results, for the first time, indicated a difference in total relative day-time export due solely to spectral quality at a very similar photosynthetic rate. It is important to note that the light intensity of the medium photosynthetic rate experiment falls within the exponential phase of the light response curve (Lanoue et al., 2017). During this phase, changes in light intensity and quality have the largest impact on a plant’s carbon status.

**FIGURE 8:**
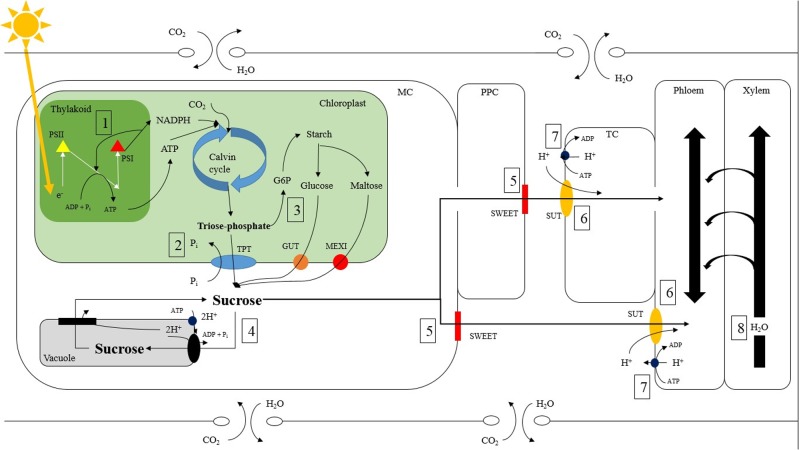
Potential sites of carbon and water regulation which can be affected by light intensity and quality within a tomato source leaf. ATP is produced via the light reactions through the movement of electrons between photosystem II (yellow triangle) and photosystem I (red triangle) allows for the conversion of CO_2_ to triose phosphate via the Calvin cycle **(1)**. Triose phosphate is then moved out of the chloroplast into the mesophyll cell (MC) via the anti-port mechanism of the triose phosphate/phosphate translocator (TPT; blue oval) where it is converted to sucrose (**2**; Stitt et al., 1987; Huber and Huber, 1990a; Walters et al., 2004). Triose phosphate can also be made into starch in a multiple step process. Starch, which is a storage molecule in tomatoes, can be broken down into either maltose or glucose and transported into the MC via a maltose excess1 transporter (MEXI; red circle) or glucose transporter (GUT; orange circle) respectively (**3**; Schleucher et al., 1998; Niittylä et al., 2004). Both maltose and glucose can then be converted to sucrose in the MC. Sucrose is then able to proceed via multiple pathways. Sucrose can enter the vacuole via an anti-port tonoplast membrane located H^+^/sucrose transporter (black oval) and conversely leave the vacuole via a tonoplast membrane located H^+^/sucrose symport (black rectangle) (**4**; Schulz et al., 2011; Etxeberria et al., 2012). Sucrose can also move into the apoplast via the ‘sugars will eventually be exported transporter’ (SWEET; red rectangle) directly from the MC or by first symplastically diffusing into the phloem parenchyma cell (PPC) (**5**; Chen et al., 2012; Feng et al., 2015). The mechanism of sucrose efflux via SWEET is currently speculated to be bidirectional uniport, however lacks concrete evidence (Chen et al., 2015). Once in the apoplast, sucrose enters the phloem directly or the transfer cell (TC) then enters the phloem symplastically. Entering the phloem directly or the TC is catalyzed by a co-transport H^+^/sucrose transporter (SUT; orange oval) (**6**; Riesmeier et al., 1992). ATPase enzymes (dark blue circle) are responsible for maintaining H^+^ gradients across membranes and are usually found close to enzymes using H^+^ symport or anti-port mechanism **(7)**. The movement of water between the xylem and phloem has also been proposed to affect carbon export rates (**8**; Smith and Milburn, 1980; Windt et al., 2006; De Swaef et al., 2013; Nikinmaa et al., 2013).

Furthermore, there was an increase in relative export during the morning hours of the medium photosynthetic rate experiment under B, RB, and O compared to W, RW, R, and G (**Figures 2G–I**). Interestingly, B and RB had ∼98 and 28% B wavelength composition respectively, while W, RW, R, and G had ∼9, 12, 0.4, and 2% B wavelength composition respectively (Supplementary Table 1). Blue light activates cryptochrome (CRY) which is a known regulator of the circadian clock (Somers et al., 1998; Chen et al., 2004). The pathways and mechanisms controlling export, from sub-cellular to the tissue level, involve many photoreceptors and sites of regulation other than those affecting C-fixation. The increased relative export during the morning hours of the medium photosynthetic rate experiment under B and RB could be due to activation of CRY. This could induce cyclic electron flow (**Figure 8-1**; Finazzi et al., 2002; Munekage et al., 2004; Shapiguzov et al., 2010) increasing ATP production needed in apoplastic phloem loading or expression levels of important enzymes and transporters facilitating export (**Figures 8-2**, 8-5, **8-6**).

Interestingly, when cucumbers were grown under LEDs with varying R:B ratios, plants grown under 100% B light had the highest photosynthesis to specific leaf mass ratio (Hogewoning et al., 2010). Moreover, carbohydrate analysis determined that leaves grown under 100% B had more sucrose and less starch (Hogewoning et al., 2010). Thus, having a high amount of sucrose at the end of the photoperiod coupled with a high photosynthesis to specific leaf mass ratio indicates higher export under B light consistent with findings presented in **Figures 4B**, **5B**.

Leaves under the O LED, also had high relative export (**Figure 4B**). The O light contained only 0.67% of PAR within the B region (Supplementary Table 1). To our knowledge, there is no evidence linking O wavelengths to carbon export directly. However, O light does bring about changes in tomato plant and leaf morphology during acclimation (Liu et al., 2011, 2012). Interestingly, O wavelengths are major spectral components provided by high pressure sodium luminaries that have been utilized in research and commercial controlled environments. The high absorption of B and O light by pigments and molecules associated with the photosystems (e.g., carotenoids), are involved in energy trapping and therefore may increase cyclic energy transduction facilitating phloem loading and export in the light (**Figure 8-1**; Finazzi et al., 2002; Munekage et al., 2004; Shapiguzov et al., 2010).

Our results indicated an increased rate of C-export from leaves exposed to B or O LEDs only at the medium photosynthetic rate (**Figures 2**, **4B**, **5B**). Hartt (1966) indicated that the light saturation point for sugar export in a sugarcane leaf was much less than the saturation point of photosynthesis. Therefore, during high photosynthetic rate export experiments, the apoplastic phloem loading pathway of tomatoes may be at or close to the light saturation point and thus not be altered by different wavelengths of light. This is consistent with the decrease in relative export as the photosynthetic rates increase (**Figure 5B**). Furthermore, Windt et al. (2006) have hypothesized that there may be upper and lower limits to the rate of carbon export within the phloem. At low light levels, such as those during the low photosynthetic level export experiment, the rate of sucrose production may be slower than the rate of export not allowing for the discrimination between spectral qualities, thus indicating a lower boundary to C-export.

### Linking Transpiration and C-Export

The link among phloem and xylem osmotic pressures, stomatal conductance, transpiration rates and carbon transport is poorly understood (Smith and Milburn, 1980; Windt et al., 2006; De Swaef et al., 2013; Nikinmaa et al., 2013). As indicated by Johnson et al. (1992), an increase in leaf transpiration should increase the turgor potential gradient between the source and sink, leading to increased C-export. Results in **Figures 1–3**, **7** confirm that, in general, a higher leaf transpiration rate increased absolute C-export rates. However, as observed in **Figure 7**, both stomatal conductance and transpiration rates reached a maximum mid-day and declined thereafter following an inherent circadian rhythm unaffected by wavelength (Dodd et al., 2004), similar to whole plant patterns determined by Lanoue et al. (2017). Thus, the entrained circadian rhythm of stomatal function controlling daily transpiration rates is not observed in C-export patterns.

Exposing leaves to light of different spectral quality can greatly complicate attempts to relate phloem export and xylem water potential. For example, during the high photosynthetic rate experimentation, the C-fixation rate was initially ∼12 μmol m^-2^ s^-1^ (**Figure 1**). While having similar photosynthetic rates, leaves illuminated with B and R LEDs produced different stomatal conductance values and transpiration rates while producing similar export rates throughout the day (**Figures 1E**, **7**). Of note, **Figures 5B**, **7G–I**, show that transpiration under O and G LEDs was lower than that under the B and R while still producing high rates of photosynthesis and export.

Even though transpiration rates and export may be strongly linked in woody species, it has been noted that this relationship may not be as strong for herbaceous species such as tomato (Nikinmaa et al., 2013). Illuminating tomato leaves with different spectral quality resulted in similar carbon export rates, but stomatal conductance and transpiration rates varied due to wavelength specific control of stomatal function (Kinoshita et al., 2001; Lanoue et al., 2017; **Figure 7**). Thus, we caution that the link between transpiration and C-export may be more complex than previously thought. Nevertheless, knowledge related to the interplay between water and C-movement within a plant and how they are affected by environmental stimuli, is needed to develop a better understanding of source/sink relationships during crop production.

### Implications

The pathway of carbon export is complex, involving numerous cells, enzymes, and transporters all of which are involved in water and carbon transport and leaf homeostasis (**Figure 8**; Stitt et al., 1987; Riesmeier et al., 1992; Sauer et al., 2004; Walters et al., 2004; Hackel et al., 2006; Sauer, 2007; Chen et al., 2012; Lemoine et al., 2013; Feng et al., 2015). Enzymes and transporters such as triose phosphate translocator (TPT; **Figure 8-2**; Stitt et al., 1987), sucrose phosphate synthase (SPS; **Figure 8-4**; Stitt et al., 1987; Huber and Huber, 1990a), ‘sugar will eventually be export transporter’ (‘SWEET’; **Figure 8-5**; Chen et al., 2012; Feng et al., 2015), and sucrose transporter (SUT; **Figure 8-6**; Riesmeier et al., 1992) provide potential sites of regulation which are susceptible to environmental stimuli. For example, SPS, a crucial enzyme involved in sucrose production has been observed to be affected by light intensity and quality via phosphorylation (Huber and Huber, 1990a,b; Tepperman et al., 2004; Suetsugu et al., 2014; Shi et al., 2016).

It is unlikely that differences in export at a medium photosynthetic rate are due solely to illumination with different spectral quality (**Figure 5B**). If spectral quality were the sole reason for differences in export, one would expect that export would be affected by light quality at both high and low photosynthetic rates as well (**Figure 5B**). It is more probable that an inter-play between spectral quality and intensity can explain the results seen (**Figure 5B**). Under high light intensity, the process of light absorption via the antenna complex is saturated and not able to differentiate between spectral qualities (**Figure 8-1**). During low irradiance levels, plants struggle to capture enough light needed to sustain growth and thus will utilize whichever spectrum is available in a sufficient manner (**Figure 8-1**). Thus, it is in this middle light level in which sufficient light is available to sustain energy production but below saturation rate in which processes such as energy transduction can be influenced by light quality.

For example, cyclic vs. linear electron flow in the thylakoid is known to be regulated by light (**Figure 8-1**; Finazzi et al., 2002; Munekage et al., 2004; Shapiguzov et al., 2010). Blue light alters the ratio of cyclic to linear electron flow in favor of the cyclic pathway allowing for more ATP production (**Figure 8-1**; Shapiguzov et al., 2010; Yamori and Shikanai, 2016). A higher energy state in cells that are sites of photo-assimilate movement could facilitate phloem loading. It is crucial to identify potential direct and/or indirect regulation points of carbon and water movements which could individually or holistically be responsible for the differences in day-time export. Doing so can provide insight into the inter-play between light intensity and quality affecting an under-explored area of plant science and provide interesting nucleation points for further research.

Wavelength specific alterations to export rates may indicate a form of coping mechanism of leaves within a dense canopy, such as those found in a natural forest or a commercial greenhouse, when environmental changes occur rapidly, such as those resulting from sun flecks or cloud cover (De Castro, 2000; Wiman and Gaydarova, 2008; Hertel et al., 2011). Specifically, within greenhouse production, this study with LEDs will contribute to optimizing artificial lighting regimes to maximize commercial yield. For example, inner-canopy LED lighting will increase not only photosynthesis but also carbon export which may lead to increases in biomass production and yield within vining greenhouse crops (Hao et al., 2012; Jokinen et al., 2012; Gomez et al., 2013; Gomez and Mitchell, 2014). For the development of commercially applicable artificial lighting, we show that O and G LEDs maintain high WUE, photosynthesis, and export, as well as the more popular R and B LEDs, and thus should be evaluated.

## Conclusion

In summary, the implications of quantifying day-time carbon export patterns show that an important, fundamental, pathway (C-export) connecting source and sink tissue, can be sustained throughout the photoperiod by wavelength specific LEDs, including O and G (**Figure 5**). Importantly, under all LED treatments, it was determined that day-time C-export is much greater than night-time export and similar patterns of export were observed. Significantly, at a medium photosynthetic rate, B and O LEDs produced an increase in day-time export rates compared to those of the W and RW illuminated leaves. The correlation between photosynthesis and export under all wavelengths was high (*r* = 0.91), consistent with previous literature. There are many sites of regulation controlling C-metabolism and H_2_O status within the leaf. Understanding the effect of light intensity and spectral quality on the fundamental C-export pathway is central to the understanding of source leaf function in both natural and controlled environment production systems.

## Author Contributions

JL, EL, and BG were involved in experimental design and manuscript preparation. JL performed the experiments and completed data analysis.

## Conflict of Interest Statement

The authors declare that the research was conducted in the absence of any commercial or financial relationships that could be construed as a potential conflict of interest.
