# Supplementary material for: Effects of Light Quality and Intensity on Diurnal Patterns and Rates of Photo-Assimilate Translocation and Transpiration in Tomato Leaves
Source: Front Plant Sci. 2018 Jun 4;9:756. doi: 10.3389/fpls.2018.00756 (PMC5994434; doi:10.3389/fpls.2018.00756)
Supplement: Supplementary file 1 [file Data_Sheet_1.docx]

**Supplementary Figures and Tables**


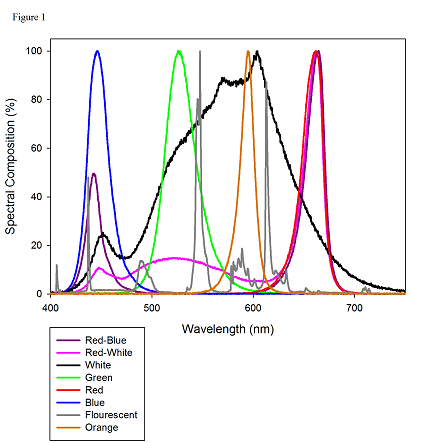


Supplementary Figure 1: PAR spectrum of compact fluorescent lights within the Biochambers used to grow the plants specifically for the ^14^CO_2_ leaf studies in which different PAR-38 LED floodlights from LSGC were tested. The PAR spectra of each of the PAR-38 LED floodlights show the wavelength composition generated by W, RB, RW, R, B, O, and G lights. Each light spectrum was determined using a spectroradiometer (Flame Spectrometer, Ocean Optics, Dunedin, FL, USA). Spectral composition (% of PAR) of each light are in Supplementary Table 1.

Supplementary Table 1: Percentage of blue, green, yellow, orange, and red wavelengths within the fluorescent growth light and each of the PAR-38 LED floodlights used to measure gas exchange and export. Percentages are based on the spectra provided in Figure 1.

|  | **Wavelength composition (% of PAR)** | **Blue**  **(400-495nm)** | **Green (495-570nm)** | **Yellow (570-590nm)** | **Orange (590-620nm)** | **Red**  **(620-750nm)** |
| --- | --- | --- | --- | --- | --- | --- |
| **Growth light** | Fluorescent | 15.78 | 41.44 | 8.49 | 24.22 | 10.13 |
| **PAR-38 floodlights** | White | 8.85 | 36.27 | 13.48 | 20.80 | 21.48 |
|  | Red-Blue | 27.96 | 0.31 | 0.09 | 0.97 | 70.67 |
|  | Red-White | 11.68 | 22.61 | 3.46 | 3.77 | 58.75 |
|  | Red | 0.36 | 0.35 | 0.14 | 1.54 | 97.62 |
|  | Blue | 98.26 | 1.06 | 0.09 | 0.13 | 0.47 |
|  | Orange | 0.67 | 4.13 | 29.67 | 64.45 | 1.62 |
|  | Green | 2.03 | 94.14 | 2.69 | 0.85 | 0.61 |
